# Optical Genome Mapping Reveals Genomic Alterations upon Gene Editing in hiPSCs: Implications for Neural Tissue Differentiation and Brain Organoid Research

**DOI:** 10.3390/cells13060507

**Published:** 2024-03-14

**Authors:** Lucia Gallego Villarejo, Wanda M. Gerding, Lisa Bachmann, Luzie H. I. Hardt, Stefan Bormann, Huu Phuc Nguyen, Thorsten Müller

**Affiliations:** 1 Department of Molecular Biochemistry, Ruhr-University Bochum, 44801 Bochum, Germany; lisa.bachmann@ruhr-uni-bochum.de (L.B.); luzie.hardt@ruhr-uni-bochum.de (L.H.I.H.); stefan.bormann@ruhr-uni-bochum.de (S.B.); 2 Department of Cytology, Institute of Anatomy, Ruhr-University Bochum, 44801 Bochum, Germany; 3 International Graduate School of Neuroscience, Ruhr-University Bochum, 44801 Bochum, Germany; 4 Department of Human Genetics, Ruhr-University Bochum, 44801 Bochum, Germany; wanda.gerding@ruhr-uni-bochum.de (W.M.G.); huu.nguyen-r7w@rub.de (H.P.N.); 5 Institute of Psychiatric Phenomics and Genomics (IPPG), University Hospital, LMU Munich, 80336 Munich, Germany; thorsten.t.mueller@ruhr-uni-bochum.de

**Keywords:** optical genome mapping, human induced pluripotent stem cells (hiPSC), cerebral organoids, CRISPR, gene editing, structural variants (SVs), copy number variants (CNVs), chromosomal aberrations, neurodevelopment

## Abstract

Genome editing, notably CRISPR (cluster regularly interspaced short palindromic repeats)/Cas9 (CRISPR-associated protein 9), has revolutionized genetic engineering allowing for precise targeted modifications. This technique’s combination with human induced pluripotent stem cells (hiPSCs) is a particularly valuable tool in cerebral organoid (CO) research. In this study, CRISPR/Cas9-generated fluorescently labeled hiPSCs exhibited no significant morphological or growth rate differences compared with unedited controls. However, genomic aberrations during gene editing necessitate efficient genome integrity assessment methods. Optical genome mapping, a high-resolution genome-wide technique, revealed genomic alterations, including chromosomal copy number gain and losses affecting numerous genes. Despite these genomic alterations, hiPSCs retain their pluripotency and capacity to generate COs without major phenotypic changes but one edited cell line showed potential neuroectodermal differentiation impairment. Thus, this study highlights optical genome mapping in assessing genome integrity in CRISPR/Cas9-edited hiPSCs emphasizing the need for comprehensive integration of genomic and morphological analysis to ensure the robustness of hiPSC-based models in cerebral organoid research.

## 1. Introduction

Among gene editing tools, the CRISPR system has been the most extensively used in mammalian cells due to its simplicity, efficiency, and low cost [1]. CRISPR-driven DNA manipulation has been optimized for use in research by combining two primary components: the Cas enzyme and a guide RNA sequence that directs the enzyme to a highly specific region of the genome [2]. There are several CRISPR/Cas systems [3]; the type II CRISPR/Cas9 enzyme from *Streptococcus pyogenes* is the most widely used. Upon recognition of the target DNA sequence, the Cas9 enzyme induces a double-strand break (DSB) at the targeted locus [4], which activates the cell’s endogenous DNA repair mechanisms, either non-homologous end joining (NHEJ) or homology-directed repair (HDR). Specifically, the HDR pathway can be used to introduce precise modifications or exogenous kilobase-scale long DNA sequences [5]. A promising application is the integration of CRISPR/Cas9 and pluripotent stem cell technology for the generation of “disease-in-a-dish” research models to explore disease-causing mutations [6] or for the development of cellular therapies.

A major concern of CRISPR/Cas as a gene therapy tool is the presence of off-target effects that could potentially result in unintended mutations, such as large genomic deletions and rearrangements, which are known to occur as a consequence of DSBs [7]. These alterations might generate genetic mutations with unknown consequences for the patient. Although multiple efforts have been undertaken to increase the specificity of Cas enzymes [8], off-target activity is still a side effect that occurs. Existing off-target detection tools [9] do not yet fulfill the expected requirements regarding sensitivity, specificity, whole genome applicability, and high throughput implementation. Numerous web-based prediction tools can only be used to forecast high-risk DNA sites, whereas the modification type must be analyzed, e.g., by PCR amplification and sequencing. Whole genome sequencing allows for the identification of off-target nuclease activity genome-wide, but the application is limited by its high cost and the dominance of unedited genomic DNA data [9]. 

The increasing need for genome-wide screening tools is met by optical genome mapping techniques including features of conventional karyotyping on the genome-wide level with high resolution. Unlike karyotyping, the analysis is based on the preparation of native high-molecular-weight DNA derived from the sample and is not influenced by the cell line cultivation and preparation method [10]. Thus, optical genome mapping not only enables the detection of most major structural rearrangements that can also be detected by karyotyping but also of minor variants that could be missed by other technologies on a genome-wide approach [11]. 

Within this work, we used optical genome mapping on cerebral organoids to assess the genome integrity of two hiPSC lines after CRISPR/Cas9-mediated gene editing. The application of this technique revealed the presence of genomic aberrations that were not underscored by morphological assessments. Moreover, we used cerebral organoids to study the potential consequences of these genomic alterations. Our results confirmed the vulnerability of genomic DNA to gene editing and highlighted the relevance of optical genome mapping as novel quality assessment for the control of genetic engineering.

## 2. Materials and Methods

### 2.1. Human Induced Pluripotent Stem Cell (hiPSC) Cultures

Two hiPSC lines (10-C (BIONi10-C) and PSEN1 +/+ (BIONi010-C-29)), kindly provided by Bioneer [12], (https://bioneer.dk/, accessed on 10 January 2024) were routinely cultured in StemFlex™ medium (Gibco™, Thermo Fisher Scientific, Waltham, MA, USA) with 1% Antibiotic/Antimycotic (Gibco™, Thermo Fisher Scientific) in 35 mm Geltrex™ (Gibco™, Thermo Fisher Scientific)-coated dishes at 37 °C and in a 5% CO_2_ humidified atmosphere. Cells were checked daily using a Leica DMI 4000 B Light Microscope. StemFlex™ (Gibco™, Thermo Fisher Scientific) medium change was performed every 1–2 days and the cells were split before they reached 70–80% confluency.

### 2.2. Homology-Directed Repair (HDR) CRISPR/Cas9 Knock-In (KI) Approach

Firstly, a gRNA was designed to target the N-terminus of the *LMNB1* gene (CgGGGGTCGCAGTCGCCATGGCG). Based on the gRNA sequence, to obtain an RNP complex, custom synthetic Alt-R^®^ CRISPR/Cas9 crRNA, and the corresponding tracrRNA to form the sgRNA complex were purchased from Integrated DNA Technologies (IDT) together with the recombinant Cas9 protein. The plasmid for tagging the N-terminus of the human *LMNB1* gene with RFP (Addgene, Watertown, MA, USA, LMNB1-mTagRFP-T, #114403) and GFP (Addgene, LMNB1-mEGFP, #87422) were purchased from Addgene and their sequence was validated by Sanger sequencing. To generate both PS1-LMNB1_GFP and LMNB1_RFP hiPSCs, the cells were detached with TrypLE™ (Gibco™, Thermo Fisher Scientific) before they reached 70% confluence and nucleofected using the 4D-Nucleofector™ X Unit (Lonza, Basel, Switzerland) with the CB-150 pulse according to the manufacturer’s instructions. Cells were mixed with the nucleofector solution and nucleofector supplement (P3 Primary Cell Kit, Lonza) followed by the Alt-R^®^ Cas9 Electroporation Enhancer (IDT), the RNP complex and 0.8 µg of the HDR-Plasmid. Afterward, cells were cultivated in StemFlex™ (STEMCELL™ Technologies, Vancouver, BC, Canada) culture medium supplemented with ROCK inhibitor (Rho-kinase inhibitor, Y-27632; STEMCELL™ Technologies) and 0.1% Alt-R^®^ HDR enhancer (Integrated DNA Technologies, Coralville, IA, USA) and monitored daily using an Olympus IX51 inverted microscope. Fluorescent signal was first observed 72 h post-transfection.

### 2.3. Fluorescence-Activated Cell Sorting (FACS) and Sanger Sequencing

7 to 10 days post nucleofection, cells were detached using TrypLE™ (Gibco™, Thermo Fisher Scientific) and centrifuged at 300× *g* for 5 min. The pellet was re-suspended in DPBS (−/−) (Gibco™, Thermo Fisher Scientific) at a concentration below 100,000 cells/mL. Cells were separated by FACS using the MoFlo Astrios Eq Cell Sorter (Beckman Coulter, Brea, CA, USA), adjusting the settings to obtain a uniform pool of LMNB1_RFP-positive (LMNB1_RFP) and LMNB1_GFP-positive (PS1-LMNB1_GFP) hiPSCs with high fluorescence. More than 3000 cells were sorted in one well of a with Geltrex™ (Gibco™, Thermo Fisher Scientific)-coated 96-well plate equilibrated with 100 μL StemFlex™ (Gibco™, Thermo Fisher Scientific) and 0.1 μL ROCK Inhibitor (Rho-kinase inhibitor, Y-27632; STEMCELL™ Technologies). Correct KI integration was further confirmed by hiPSC DNA extraction, polymerase chain reaction amplification (using primers LMNB1_RFP_fw gtgcttctccgttcctctaa, LMNB1_RFP_rev gtctgtggtccacatagtaga, LMNB1_RFP_downstream_fw caacaccgagatgctgta, and LMNB1_RFP_downstream_rev cctggtctactatctgcaca; LMNB1_GFP_fw ggtgcttctccgttcctctaaac, LMNB1_GFP_rev tgaagtcgatgcccttcagctc, LMNB1_GFP_downstream_fw CGACGGCAACTACAAGAC LMNB1_GFP_downstream_rev cctggtctactatctgcaca), and Sanger sequencing.

### 2.4. Growth Rate Determination

To determine the growth rate of gene-edited and unedited hiPSCs lines, cells under 70% confluence were dissociated using TrypLE™ (Gibco™, Thermo Fisher Scientific), following established protocols. Subsequently, they were resuspended in StemFlex™ medium (Gibco™, Thermo Fisher Scientific) and the cell count was assessed using a Neubauer Chamber (Marienfeld, Lauda-Königshofen, Germany). The suitable volume yielding a total of 300,000 cells, was centrifuged at 300× *g* for 5 min. The resulting pellet was then resuspended in StemFlex™ (Gibco™, Thermo Fisher Scientific) supplemented with 0.1% RI (Y-27632, STEMCELL Technologies) and seeded onto Geltrex™ (Gibco™, Thermo Fisher Scientific)-coated dishes. Repetition of this process allowed the determination of cell growth within 48 hours. The collected data was presented graphically, and differences were statistically evaluated using a two-sample Student’s *t*-test. Results with *p* < 0.05 were deemed statistically significant.

### 2.5. Cerebral Organoid Generation

Gene-edited hiPSC lines (PS1-LMNB1_GFP and LMNB1_RFP) were used to develop organoids following the protocol published by Lancaster and Knoblich [13] with minor modifications, as described in Bachmann et al., 2022 [14]. Briefly, on days 1 and 2 after seeding, the EBs were monitored for their appearance and size using a light microscope. If the EBs showed blurred edges or a diameter < 300 μm, half of the medium was aspirated and exchanged with 150 μL fresh hESC medium containing 4 ng/mL bFGF (Peprotech, Cranbury, NJ, USA) and 50 μM ROCK inhibitor (Rho-kinase inhibitor, Y-27632; STEMCELL™ Technologies). On day 3, the medium was changed to hESC medium without supplements. On day 6, the medium was changed to neural induction medium and then changed every other day until day 11 or 12, when EBs were embedded in Matrigel^®^ (GFR) Basement Membrane Matrix (Corning^®^, Corning, NY, USA) droplets. Afterward, organoids were kept for long-term culture.

### 2.6. Immunofluorescence Staining and Imaging

Fixation, sectioning, and immunofluorescence staining of cerebral organoids was performed as described before in Bachmann et al., 2022 [14]. The CO sections were thawed in PBS, permeabilized and blocked with 0.1% Triton X-100 (Carl Roth, Karlsruhe, Germany) and 5% normal goat serum (Southern Biotech, Birmingham, AL, USA) in PBS for 1 h. Primary antibodies were diluted in 0.1% Triton X-100 (Carl Roth) in PBS (βIII-tubulin [2G10]: ab78078, Abcam, 1:500; MAP2: M4403, Sigma–Aldrich^®^, St. Louis, MO, USA, 1:500; PAX6: 901301, BioLegend^®^, San Diego, CA, USA) and incubated on the CO sections in a humidified chamber at 4°C overnight. Next day, the sections were washed twice and incubated with the secondary antibody Goat Anti-Mouse IgG H&L (Alexa Fluor^®^ 488) and Goat Anti-Rabbit IgG H&L (Alexa Fluor^®^ 555) (A11001, A32732, Thermo Fisher Scientific) diluted 1:1000 in 0.1% Triton X-100 (Carl Roth) in a humidified chamber for 45 min. The sections were washed twice and incubated with 0.001 mg/mL DAPI or 0.001 mg/mL Hoechst 33342 (Invitrogen™, Thermo Fisher Scientific) for 15 min. Samples were imaged with a Leica TCS SP8 confocal microscope system equipped with a 10 × (0.3 NA) or 100× oil objective (1.4 NA) and a 405 nm and white light laser with excitation wavelengths of 405 nm (DAPI/Hoechst33342), 488 nm (eGFP, Alexa Fluor^®^ 488), and 551 nm (RFP, Alexa Fluor^®^ 555) and emission wavelengths at ranges of 410–463 nm (DAPI/Hoechst33342), 493–592 nm (eGFP, Alexa Fluor^®^ 488), and 556–701 nm (RFP, Alexa Fluor^®^ 555). For secondary antibody-couple fluorophores, hybrid detectors were used, whereas a photomultiplier tube detector (PMT) was used for Hoechst 3342 and DAPI. The images were recorded in a sequential scan speed of 600 Hz. Images were processed with the LAS X software 3.5.7.23225 (Leica, Leitz, Wetzlar, Germany). Alternatively, images were captured using a fully automated bright-field and fluorescence Keyence BZ-X800E microscope, which was equipped with a 10× dry objective.

### 2.7. DNA Isolation and Optical Genome Mapping

Cells were collected and prepared according to the manufacturers’ instructions using a Bionano SP Blood & Cell Culture DNA isolation kit (Bionano Genomics, San Diego, CA, USA) and a Direct Label and Stain kit (DLS kit including Direct labeling enzyme DLE-1 enzyme, Bionano Genomics, San Diego, CA, USA). Labeled DNA was run on a Bionano Genomics Saphyr^®^ System using a Bionano Genomics Saphyr Chip G2.3 [15]. Bionano Access 1.7.1.1 and Bionano Solve 3.7 were utilized for further data processing and visualization. The manufacturer’s quality metrics recommendations and 80× coverage (downsampling was performed where necessary) were achieved for all samples enabling downstream analyses via de novo pipelines. Using this analysis, labeling patterns between the cell line and a GRCh37/19 reference genome map were compared. Identified SVs were filtered using the default settings and a filter for structural variants (SVs) in ≤1% of the control database based on a human population of 179 ethnically diverse DLE-1 labeled genomes that were published by the manufacturer [16]. Whether SVs might be located in highly variable regions was analyzed using the rare variant hg19 DLE-1 SV mask. To analyze SVs and CNVs that occurred before and after gene editing in the hiPSC lines studied, duo analysis was applied as the second analysis step using Bionano Access software 1.7.1.1. Here, the variant annotation pipeline extracted information on whether the cells were sample-specific; this means that they were analyzed whether variants were found in the assemblies and molecules of a selected (control) sample, which in our case was an hiPSC line before gene editing in our laboratory. Duo analysis enables the visualization of SVs and CNVs that can be found exclusively in the sample and not in the (control) sample used for normalization.

## 3. Results

### 3.1. Generation of Test Model: Gene Edited Cell Lines with Alzheirmer’s Disease (AD) Specific Mutation for the Differentiation of Cerebral Organoids

**Figure 1:**
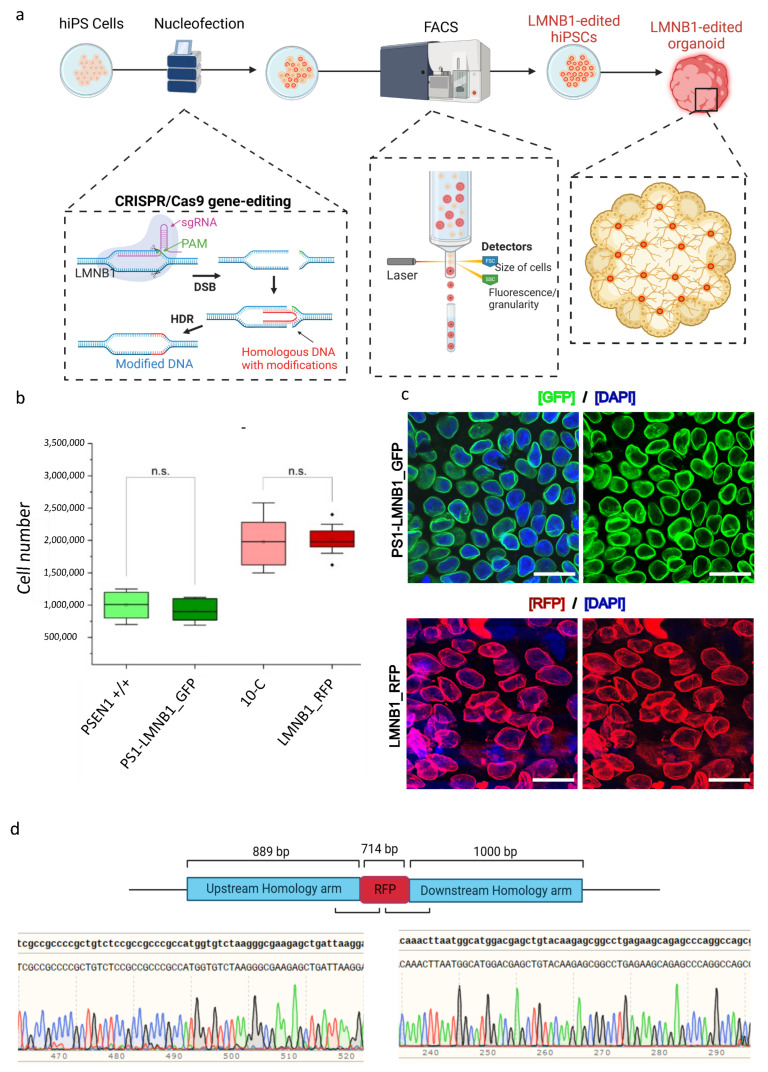
Development and characterization of two CRISPR/Cas9-labeled FACS-enriched hiPSC populations. (**a**) Schematic representation of the workflow. HiPSCs were nucleofected with CRISPR/Cas9, sgRNA targeting the *LMNB1* gene and a plasmid containing the desired modification. HiPSCs that successfully included the knock-in were separated by fluorescence-activated cell sorting (FACS) and further used for cerebral organoid development. (**b**) Box chart depicting the gene-edited and the respective non-edited parental hiPSC lines’ comparable growth rates, represented by the maintenance of a stable cell number 48 h after passage. Significance *p* < 0.05 according to standard Student’s *t*-test. n.s: not significant. (**c**) Representative confocal microscopy images of LMNB1 RFP/GFP FACS-enriched cell populations showing GFP+ and RFP+ cells with homogeneous fluorescence intensity levels and perinuclear lamin B1 phenotype. Scale bars: 25 µm. (**d**) Sanger sequencing data obtained after analysis of DNA of LMNB1_RFP hiPSC showing the correct knock-in of the RFP in frame with the *LMNB1* sequence.

We aimed to differentiate a mixed cerebral organoid model using human induced pluripotent stem cells (hiPSCs) by combining two distinct cell lines in equal proportions to generate a single brain-like structure [14]. Given our lab’s main focus on understanding neurodegenerative disorders such as Alzheimer’s disease (AD), where presenilin-1 (PSEN1) mutations play a central role, we used two commercially available hiPSC lines: 10-C and the isogenic counterpart carrying the E280A mutation in the *PSEN1* gene (PSEN1 +/+) [12]. To distinguish between the two distinct genotypes of the cell lines within the same organoid, we created two reporter hiPSC lines by using CRISPR/Cas9 to fluorescently tag the lamin B1 protein, as its ubiquitous expression allows for the identification of the cells regardless of their differentiation state. Moreover, the characteristic perinuclear phenotype of this protein enables an initial microscopic assessment of the success of the gene editing strategy. Therefore, we designed a CRISPR/Cas9 HDR-based gene editing approach, following previously described protocols [17], to tag the N-terminal region of the lamin B1 protein with a green or a red fluorescent protein (RFP/GFP). After transfection and fluorescent microscopy confirmation of the successful knock-in (K), two FACS-enriched populations of gene-edited cells were generated: LMNB1_RFP and PS1- LMNB1_GFP (Figure 1a).

To assess the possible effects arising from the editing process, the LMNB1_RFP and PS1-LMNB1_GFP hiPSC populations were monitored daily by microscopy regarding their growth rate and morphology, showing no differences in comparison with the unedited controls. All the hiPSC lines maintained their stem cell self-renewal capacity and morphological characteristics with roundish clear borders, high nuclei to cytoplasm ratios, and prominent nucleoli [18] and displayed healthy, undifferentiated morphologies forming compact colonies (Supplementary Figure S1a). Similarly, the gene-edited hiPSC populations displayed a stable growth rate, with no significant differences compared with the control hiPSC lines (Figure 1b).

When analyzed by fluorescent microscopy as part of the quality-control process, all studied cells showed a perinuclear GFP/RFP fluorescent ring coincident with the expected lamin B1 phenotype and cellular location. The fluorescent signal intensity was stable and maintained over the course of the culture period and after multiple passages (Figure 1c). Subsequently, Sanger sequencing was used to confirm the precise integration of the RFP/GFP cassette in frame with the N-terminal sequence of the lamin B1 protein and the absence of on-target plasmid backbone integration (Figure 1d and Supplementary Figure S1b). Taken together, the FACS-enriched populations displayed a homogenous genetic profile that pointed out a precise and successful gene-editing strategy. Moreover, imaging analysis showed consistency regarding the expected subcellular localization of the LMNB1-tagged protein and no alterations in cellular morphology or growth rate were displayed. 

### 3.2. Optical Genome Mapping as Quality Control Method after Gene-Editing

Being aware that gene-editing and long-term culture of hiPSC lines are stressing procedures that might cause the fixation of somatic mutations by selection pressure [19], we decided to further analyze the gene-edited and non-gene-edited isogenic hiPSC lines using optical genome mapping (OGM) for the identification of structural variants (SVs) and copy number variants (CNVs) on a genome-wide level.

**Figure 2:**
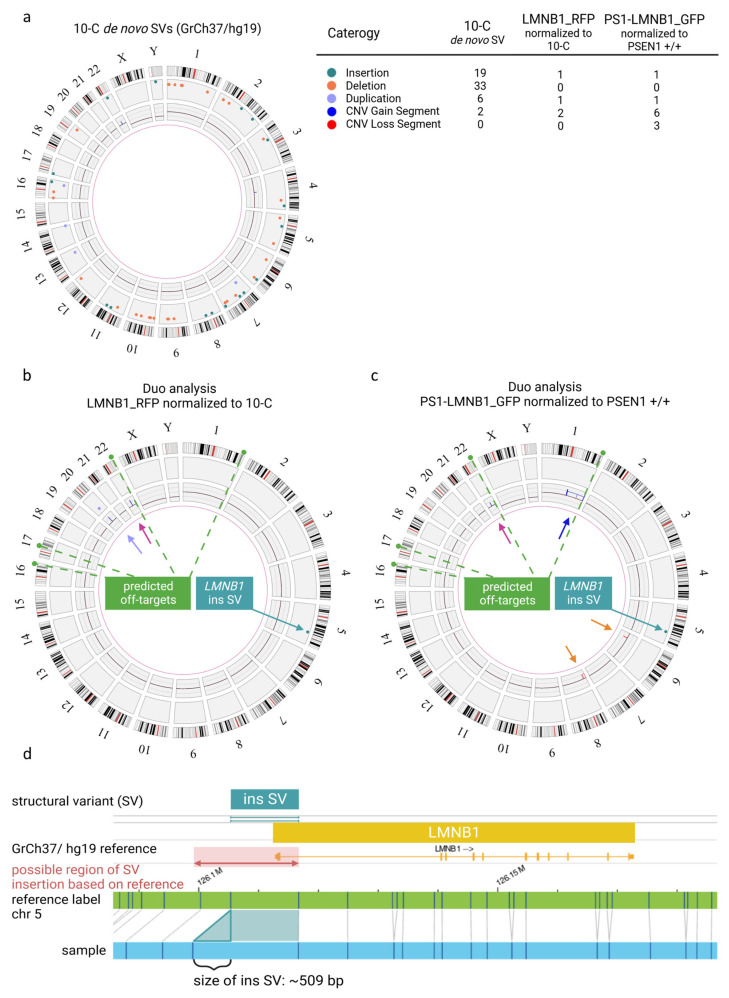
Quality assessment of genomic integrity in hiPSCs after gene editing and long-term culture using optical genome mapping. (**a**) Circos plot of 10-C hiPSC line normalized to the human genome reference GrCh37/h19 presenting multiple SVs and CNVs as detailed in the legend. (**b**) Duo analysis for LMNB_RFP hiPSC normalized to 10 C. The chromosome 22 variant (pink arrow) is detected by CNV pipeline (blue line). The variant on chromosome 20 (purple arrow) is detected by both CNV (blue line) and SV (purple dot). Modification coincident with RFP fluorophore on chromosome 5 (green arrow) is detected by SV pipeline (green dot). (**c**) Duo analysis for PS1-LMNB_GFP hiPSC normalized to PSEN1 +/+. The chromosome 22 copy number gain (pink arrow) is detected CNV pipeline (blue line). The chromosome 1 variant (dark blue arrow) is detected by CNV pipeline (blue line). Copy number losses on in chromosome 6 and 8 (orange arrows) are detected by CNV (red lines) pipeline. Modification coincident with GFP fluorophore on chromosome 5 (green arrow) is detected as SV (green dot). Predicted off-target sites by CasOFFinder tool are indicated in (**b**) and (**c**) (green dashed line). (**d**) Detail view of insertion on chromosome 5 in *LMNB1* gene.

Interestingly, when the non-edited 10-C hiPSC line was compared to a control human reference sequence (GrCh37/hg19), multiple SVs were detected. In total, in a male karyotype, 58 SVs were detected that are not present in 1% or less of the samples from the control human database provided by the manufacturer. In addition to this, two large copy number gains > 500 kb were identified (Figure 2a). These results confirmed the presence of individual variants in the hiPSC line analyzed and emphasized the need for specific controls after gene editing. Subsequently, the gene-edited LMNB1_RFP hiPSC population was analyzed normalizing the results to the non-edited isogenic 10-C hiPSC line using a duo analysis approach. As expected, almost all the SVs were present in both samples, and therefore they were no longer visualized in the circos plot for LMNB1_RFP (Figure 2b) after normalization, confirming the isogeneity of the cell lines. However, additional SVs and CNVs were detected in the gene-edited LMNB1_RFP hiPSC population. Firstly, a structural variant (insertion) with a size of about 509 bp was found on chromosome 5, in the region of the following label positions: 126105333_126116693. Considering that optical genome mapping is not a sequencing technique with base pair accuracy, this can be assumed to represent the location of the RFP fluorophore (729 bp) insertion in the N-terminal site of the *LMNB1* gene (chromosome 5). This modification was exclusively present in the gene-edited LMNB1_RFP hiPSC population (Figure 2b), confirming the correct integration of the fluorophore. In line with the cell line provider’s analysis, a copy number gain of ~1335 Megabases on chromosome 22 (22q11.23(23676187_25011672) x3~4) was detected that was present in both the edited and unedited hiPSC lines (Figure 2b, pink arrow). Interestingly, an additional variant was identified in the 20q11.21 region, presenting as both a duplication split dup (20)(q11.21q11.21) sized 529,391 kb and a CNV gain of 1.25 Mb at 20q11.21 (29651348_30901959) x3~4 (Figure 2b, purple arrow). However, this variant was located in a highly variable region that is usually masked in structural variant detection of the applied methodology and, thus, these results have to be interpreted with caution. Nevertheless, this is noticeable as this region has been previously described to be one of the most recurrent variants found in hiPSCs and to compromise some relevant cancer-related genes (Supplementary Table S1) [20].

In addition, the PS1-LMNB1_GFP population was analyzed and compared with the isogenic, commercially acquired, non-edited PSEN1 +/+ cell line. SVs analysis revealed an insertion on chromosome 5 (Figure 2c), aligning with the GFP knock-in (KI), mirroring previous findings in the LMNB1_RFP line. Likewise, the CNV analysis revealed the previously described gain on chromosome 22 (Figure 2c, pink arrow). Additionally, several copy number gains on chromosome 1 were present, which can be summarized to one large copy number gain corresponding to a trisomy 1q21.1q44(44452084_248878513) ~x3 which compromises the long arm of chromosome 1 affecting a great number of genes (Supplementary Table S1) because of its size (~204 Megabases) (Figure 2c, blue arrow). Moreover, one copy number loss of ~1.5 Megabases was detected on chromosome 6 (6p21.33(30580139_32060913) ~x1) and two copy number losses were detected on chromosome 8, which can be summarized to a deletion of about 16 Megabases in total (8p23.3p22(11805_16756263) x1) (Figure 2c, orange arrows). These changes might be crucial to the cell line’s phenotype because of potential haploinsufficiency for a high number of genes (Supplementary Table S1). Notably, analysis of the sgRNA using the Cas-OFFinder tool [21] revealed that, among the various predicted off-target regions (Supplementary Table S2), one was located within the large aberration detected by optical genome mapping on chromosome 1. However, the detected copy number change had a size of about 204 Megabases, whereas the predicted off-target site comprised 26 bases, hindering the determination of the potential correlation between the predicted off-target site and the copy number gain on chromosome 1. In contrast, the predicted off-target sites on chromosomes 21, 17 and 16 did not exhibit any detectable structural copy variants in these predicted regions.

### 3.3. Relevance of Identified Genomic Alterations for the Correct Differentiation of Neural Tissue within Cerebral Organoids

Some of the quality assessments that are routinely conducted on hiPSCs to ensure their genomic integrity after gene editing rely on morphological observation. However, within this study, gene-edited cell lines carrying significant aberrations exhibited typical stem cell morphological characteristics and did not display an acquired growth advantage. In this regard, the development of embryoid bodies (EBs) is commonly used as an additional indicator of hiPSC quality, reflecting the maintenance of the stem cell’s capacity to differentiate into neuroectodermal linage cell populations [22]. 

**Figure 3:**
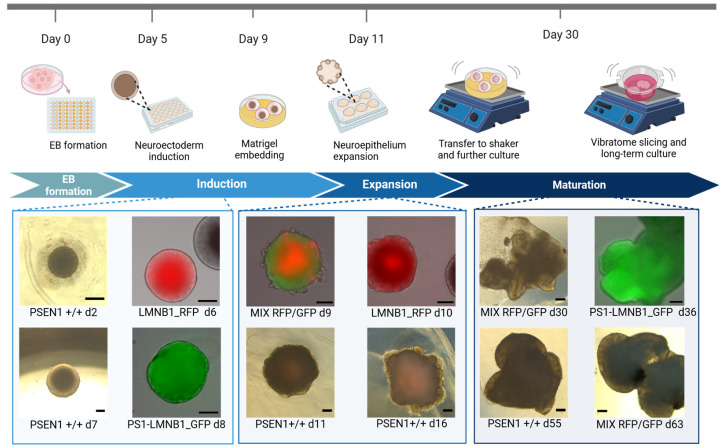
Generation of cerebral organoids from gene-edited fluorescent hiPSC populations. Schematic representation of cerebral organoid generation pipeline including representative pictures of organoids displaying characteristic morphological features. Scale bar: 200 μm.

Intrigued by the results obtained by OGM, we decided to assess whether the major genomic alterations found affected the hiPSCs’ capacity to differentiate into neural tissue, therefore evaluating the efficacy of EB development as an hiPSC quality control tool. Moreover, due to the location of the genomic alteration in regions containing genes related to major cellular functions (Supplementary Table S1), we tried to elucidate possible functional consequences of these genomic alterations on cell growth and differentiation. Therefore, cerebral organoids (COs) were generated with PS1-LMNB1_GFP and LMNB1_RFP hiPSC populations. Additionally, to minimize the potential effects caused by differences in cell culture conditions, both gene-edited lines were used together in equal proportions to differentiate mixed organoids (MIX RFP/GFP). Subsequently, the presence of characteristic features for neuroepithelium development were assessed by microscopy. Firstly, it was observed that both lamin B1-tagged hiPSC lines maintained their pluripotency and differentiation capacity as shown by their ability to develop EBs (Figure 3) with expected sizes and morphologies, recapitulating the key features predicted for these structures [23]. Moreover, the COs displayed stable expression of the lamin B1-tag protein after maturation (Supplementary Figure S2).

**Figure 4:**
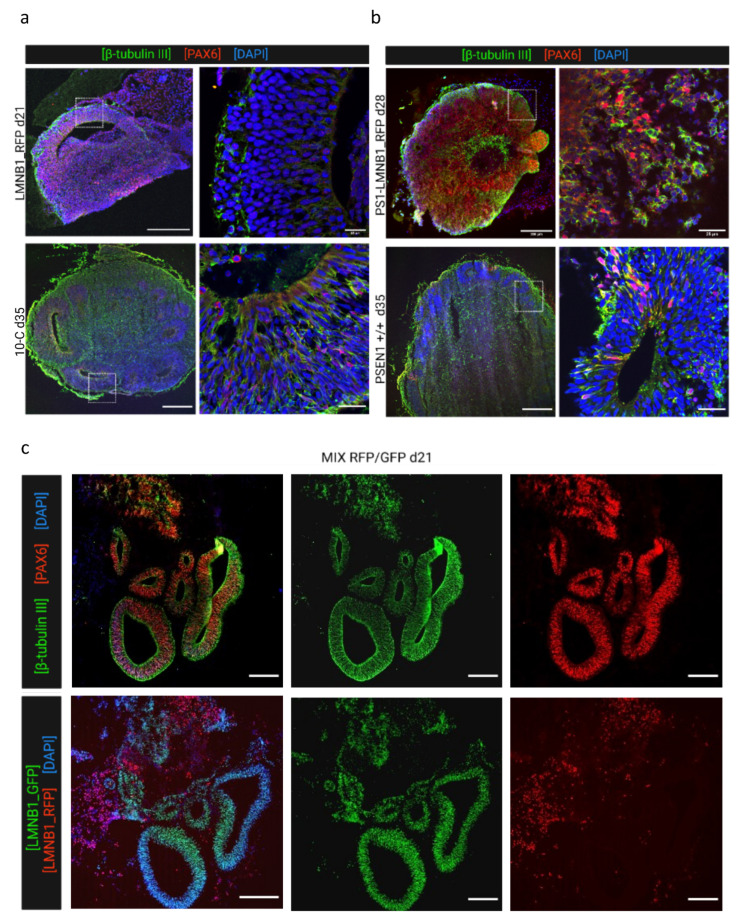
Cerebral organoids generated from gene-edited cell lines display the expected development of neural cells. (**a**) Representative images of PS1-LMNB1_GFP organoids (**upper panel**) compared with PSEN1 +/+ isogenic non-edited organoids (**bottom panel**) at day 35 immunofluorescence-stained for neural progenitors (PAX6) and immature neurons (β-tubulin III). Both organoids showed primarily neural differentiation. (**b**) Representative organoid sections from LMNB1_RFP (**upper panel**) and (**lower panel**) stained for markers of neural progenitors (PAX6) and immature neurons (β-tubulin III). The gene edited cell line showed a reduction in the number of neural progenitor areas as compared with COs generated from the non-edited isogenic cell line. (**c**) Representative immunofluorescence images of MIX RFP/GFP organoids displaying correct development of PAX6+ and β-tubulin III+ (**upper row**) and fluorescent microscopy pictures for lamin B1-tag analysis showing that progenitor areas were conformed primarily by PS1-LMNB1 hiPSCs (**lower row**). Scale bars: 200 μm (overview) and 25 μm (magnification).

Furthermore, samples were collected at various time points and analyzed by immunofluorescence regarding their cellular composition, distribution, and characteristics in comparison with the non-edited isogenic 10-C and PSEN1 +/+ hiPSC lines. PS1-LMNB1_GFP cerebral organoids analyzed at 24 days displayed multiple well-structured progenitor areas formed by neural progenitor cells (Pax-6+) organized in layers and intertwined with immature neurons (β-tubulin III+) (Figure 4a, upper panel). Furthermore, no differences could be detected when compared to the isogenic non-edited control (PSEN1 +/+), that displayed a similar morphology (Figure 4a, lower panel). Therefore, no effect derived from the major genomic alterations found by OGM was detected. Subsequently, LMNB1_RFP COs were analyzed at day 35 and compared with the non-edited 10-C COs. In this case, a reduced amount of progenitor areas in LMNB1_RFP COs could be observed, although they were comparably populated by neuronal progenitors (Pax-6+) and immature neurons (β-tubulin III+) (Figure 4b, upper panel).

Lastly, the morphological alterations observed previously were analyzed in more detail in 23-days old MIX RFP/GFP COs. These samples displayed multiple progenitor areas with a similar morphology and cellular composition to what was found in PS1-LMNB1_GFP COs (Figure 4c, upper panel). However, taking advantage of the lamin B1 fluorescent label, it was possible to differentiate that these areas were mainly formed exclusively by PS1-LMNB1_GFP hiPSCs, while the LMNB1_RFP hiPSCs were located randomly dispersed through the tissue, surrounding the GFP+ progenitor areas (Figure 4c, lower panel). These differences support previous observations regarding the LMNB1_RFP hiPSCs differentiation impairment (Figure 4a), although cell growth differences observed between cell lines in culture (Figure 1b) should be considered when analyzing these results.

In summary, genomic alterations found by OGM only induced subtle effects pointing to an impediment of neuronal induction and differentiation within the LMNB1_RFP cell line exhibiting diminished quantity of progenitor areas, irrespective of whether they were cultured alone or in combination with another cell line in mixed organoids.

## 4. Discussion

Within this study, two gene-edited fluorescent hiPSC populations were generated and monitored for the standard hiPSC quality parameters, including cell morphology and growth rate [17,18], not indicating any noticeable differences when compared to the non-gene-edited isogenic hiPSC lines (Figure 1 and Supplementary Figure S1). Despite these results, detailed genomic analysis using optical genome mapping (OGM) revealed multiple aberrations that affected a large number of genes (Supplementary Table S1) but seemed to have mild impact on the ability of hiPSCs to develop cerebral organoids (Figure 3 and Figure 4).

To avoid undesired genomic modifications in hiPSCs described to arise after gene editing [24,25,26,27], within this study, the gRNA was designed according to the established literature standards [28,29,30] and showed high quality when analyzed using the in silico tool Cas-OFFinder (Supplementary Table S2) [31,32]. Another source of genomic variability is the long-term cultivation of stem cells [33,34,35,36,37,38]. Specifically, certain steps within the hiPSC gene-editing pipeline require suboptimal culture conditions and might favor the emergence and fixation of genomic abnormalities, potentially providing a selective growth advantage to the cells [34,39,40]. Although the gene-editing pipeline fulfilled the general requirements for high quality and minimum off targets, undesired genetic aberrations were present in the gene-edited hiPSCs, as evidenced by optical genome mapping (OGM). This stresses the need for the consideration of pipeline refinements to be implemented, such as the use of Cas variants with increased efficiency [8] or the application of modulatory small molecules [41]. However, there is still not a universally applicable strategy, and the workflow must be carefully selected and adapted depending on the goal and constraints of the project.

Furthermore, it is important to consider the well-documented intrinsic genomic instability of iPSCs [42] evidenced by the multiple genetic variants found in different cell lines after prolonged culture, [33,34,43,44,45], with some of them being recurrent [46,47]. These alterations have been proposed to have a positive impact on the cells’ survival or proliferation capacity [20,48,49,50], emphasizing the need for comprehensive analyses that include techniques to assess the cells’ genomic stability and integrity, such as GTG-banding, fluorescence in situ hybridization (FISH), single-nucleotide polymorphism (SNP) microarray, comparative genome hybridization array (aCGH), and next-generation sequencing (NGS) [40,51]. However, none of the available techniques meet all requirements in terms of resolution, sensibility, and low costs and genomic control often relies mainly on GTG-banding karyotype analysis [52]. The EB development and morphological characterization of organoids is generally employed as a complementary quality control method. However, the lack of noticeable variations in the growth, morphology, or cell composition of cerebral organoids despite the significant alterations in crucial genes observed within this work (Figure 3 and Figure 4) emphasizes its limitation as a quality control method. Therefore, there is an urgent need for novel DNA-based quality control approaches to increase the reliability of the results obtained from stem cell studies excluding possible effects caused by undetected genetic alterations. Optical genome mapping has emerged as a valuable, complementary tool for genetic integrity analysis [53]. This technique presents a higher resolution than conventional chromosome analysis, allowing for the highly sensitive detection of a wide range of genetic alterations, such as homozygous and heterozygous insertions, deletions, duplications, inversions, and translocations, in a diploid genome as small as 500 bp. Moreover, the copy number pipeline exhibits a resolution of 500 kb for the detection of copy number variants, which account for a large proportion of genomic variation among individuals [54,55]. Additionally, OMG enables the whole-genome characterization of previously undetectable aberrations [53]. Therefore, it has been recently used together with complementary quality control methods to assess the genome integrity of several hiPSC lines, identifying genomic alterations induced by reprogramming [56] and CRISPR/Cas9 genome editing [57,58], some of which were previously unrecognized [59], and to quantify and characterize chromosomal instability arising at different time points during iPSC culture [60]. On the other hand, the duo analysis approach, which relies on the normalization of the genomic data to a given data, can be used to reveal changes on a genomic level in hiPSCs before and after treatment/gene editing, accounting for the intrinsic genomic characteristics of the specific cell line. This approach has already been applied in the organoid field to generate long-read genomes of great apes and characterize differences in gene expression [61]. However, this methodology has its limitations, such as its resolution [62]. Thus, although it is superior to karyotyping, breakpoints of balanced SVs located within large repetitive, unmappable regions, such as centromeres, the p arm of acrocentric chromosomes, or constitutive heterochromatin stretches [63], are difficult to analyze. Therefore, to ensure a resolution on the single base level, which is not covered by OGM, the use of complementary techniques such as next-generation sequencing and long-read sequencing is still required.

Interestingly, the results within this work suggest the potential use of cerebral organoids as a tool to assess the functional consequences of genomic alterations in hiPSCs. Thus, OGM analysis revealed a duplication on chromosome 20q11.21 within the LMNB1_RFP hiPSC population, which has been reported as a recurrent abnormality in different hiPSC lines [44,64,65,66,67]. This region contains different candidate genes associated with pluripotency and antiapoptotic effects. Specifically, *BCL2L1* has been proposed to confer cell culture adaptation advantages [20,68] promoting cell survival [43,68,69] and improving cloning efficiency [48]. Alternatively, studies on hiPSCs and EBs have reported changes in the expression of genes related to the PI3K/AKT pathway [69], which are known to be essential for stem cell proliferation and survival [70,71]. In this regard, our results did not detect an increase in the LMNB1_RFP cell line division rate that suggested a growth advantage (Figure 1b). On the other hand, the 20q gain has also been associated with impairments in ectodermal commitment and neuroectodermal differentiation in hiPSCs [72]. This might explain the maintenance of the LMNB1_RFP cells’ ability to develop EBs with normal morphological characteristics (Figure 3) that later develop a reduced amount of neural progenitor structures (Figure 4a). Similarly, the results obtained in MIX RFP/GFP organoids showing neural progenitor structures derived preferentially from PS1-LMNB1_GFP cells, and not from LMNB1_RFP cells, support the hurdle of the cell line to differentiate into neuroectodermal cell types (Figure 4c). However, this observed tendency was not further quantified and, therefore, qualitative results must be carefully interpreted. 

Other genetic aberrations recurrent in hiPSCs are located on chromosomes 12, 17, and X [73]. Interestingly, no alterations on these chromosomes were found within this work using OGM. However, a duplication on chromosome 1q, which was previously reported in gene-edited hiPSCs [33,44,74], ESCs [20,33,65,66,74], and derivatives [75,76], was detected in the PS1-LMNB1_GFP cell line. This genetic modification has been associated with an increase in proliferation rate [75], potentially due to *AKT3* overexpression [77]. Strikingly, within this study, no increase in the proliferation rate was detectable in PS1-LMNB1_GFP hiPSCs harboring the 1q duplication (Figure 1b). However, AKT3 is also remarkably enriched in neural progenitor cells of the human fetal cortex, pointing to a primary role in brain development [78], and its dysfunction has been associated with neurodevelopmental, neurodegenerative, and neuropsychiatric diseases [79,80,81]. Thus, the dysregulation of this gene might account for the tendency of PS1- LMNB1_GFP cells to preferentially develop neural progenitor areas when compared with the LMNB1_RFP cells within the MIX RFP/GFP organoid (Figure 4c). It is worth noting that the 1q region encompasses an off-target site predicted by Cas-OFFinder (Supplementary Table S2), which highlights the potential of CRISPR/Cas9 to induce significant genome rearrangements, while also underscoring the limitations of in silico tools to fully capture the complexity of CRISPR/Cas editing outcomes. The absence of alterations at other in silico-identified off-target sites also suggests that the specificity of CRISPR/Cas9 is influenced by more than just sequence homology [82,83], emphasizing the critical importance of empirical validation on in silico-predicted off-targets and the need for comprehensive genomic analysis to ensure the safety and efficacy of CRISPR/Cas9 applications.

The PS1-LMNB1_GFP cell line also exhibited deletions on Chr 6 6p21.33 (30580139_32060913) and Chr 8 (8p23.3p22(11805_16756263) x1) (Figure 2b). Mutations in 6p21.33 have been described to correlate with phenotypes causing mild intellectual disability in human patients, potentially due to the haploinsufficiency of *CSNK2B* [84,85]. This gene, which is involved in cell proliferation and differentiation [86,87], is known to play a crucial role in embryogenesis, central nervous system development, and organogenesis [88]. However, the complete deletion of *CSNK2B* in the PS1-LMNB1_GFP line, which has not been previously reported in the literature to our knowledge, did not induce observable changes in the growth (Figure 1b) or differentiation capacity, as depicted by the ability of the cell line to develop cerebral organoids with expected phenotypes and cellular compositions (Figure 4b). Similarly, alterations in other relevant genes, such as *DHX16*, which is involved in embryogenesis [89] and cellular growth and division [90] and is associated with developmental disorders [91], or *POU5F1*, which is crucial for stem cell pluripotency maintenance [92], did not severely affect the PS1-LMNB1_GFP CO phenotypes. Additionally, disruptions to *TUBB* (Supplementary Table S1) have been proposed to induce a variety of neurological phenotypes as this gene encodes the structural protein beta tubulin I, which is highly expressed in neural progenitors and postmitotic neurons during fetal brain development [93]. Although the potential effect of *TUBB* variations was not specifically assessed within this study, no major differences in immature neuronal differentiation, distribution, or morphology were found in the β-tubulin III-positive cells studied in day-28 PS1-LMNB1_GFP organoids (Figure 4b). Furthermore, the deletion detected in the 8p23.3 region, affecting the *TUSC3* gene which has been associated with a severe neurodevelopmental disorder [94,95], produced no detectable variation in the PS1-LMNB1_GFP hiPSCs’ characteristics. Thus, the potential effects of the multiple genomic alterations in cell function and neural development, only partially recapitulated by the CO phenotypes, emphasizes the need for an extensive genomic characterization of the hiPSCs used for cerebral organoid development to avoid misinterpretation of the results.

Altogether, our results reinforce the idea that adhering to gene editing and hiPSC culture recommendations is insufficient to prevent unintended genomic alterations. Therefore, establishing general quality standard requirements that include highly sensitive genomic integrity assessment techniques is crucial to ensure data viability and reproducibility, and to set a fixed standard for analyzing long-term cultured and/or genome edited stem cells and organoids.

## 5. Conclusions

Our study on human induced pluripotent stem cells (hiPSCs) and CRISPR/Cas9 gene editing emphasizes that strict adherence to existing gene editing and hiPSC standard protocols may not be sufficient to prevent unintended genomic alterations produced by gene editing strategies and long-term culture. This underscores the necessity for ongoing refinement of these techniques to reduce off-target effects. Moreover, our findings where notable genetic changes in hiPSCs did not manifest in the growth or morphology of derived cerebral organoids, highlights the insufficiency of morphological assessment as a standalone quality method and the need for establishing comprehensive quality standards that integrate highly sensitive techniques for genome analysis to ensure the viability and reproducibility of data. Incorporating advanced methods like optical genome mapping, despite its limitations in resolution, can provide a more thorough understanding of genomic integrity. The integration of these sensitive techniques with existing protocols is essential to understand the complexities of genome editing and to maintain the integrity and reliability of the research in this rapidly evolving field.

## Data Availability

OGM output data after bionano access software analysis that support the findings of our study are openly available in https://zenodo.org/records/10422172 (accessed on 12 March 2024) at URL (https://doi.org/10.5281/zenodo.10422171). OGM raw data are available upon request.

## Supplementary Materials

The following supporting information can be downloaded at: https://www.mdpi.com/article/10.3390/cells13060507/s1, Table S1: Gene list of affected genes as revealed by optical genome mapping in hiPSCs, Table S2: Potential CRISPR/Cas9 off-target sites identified by Cas-OFFinder for the designed gRNA, Supplementary Figure S1: Morphological and genetic assessment of CRISPR/Cas9-mediated LMNB1 gene editing, Supplementary Figure S2: Gene-edited cerebral organoids stable LMNB1-tag expression.
