# Supplementary material for: Optical Genome Mapping Reveals Genomic Alterations upon Gene Editing in hiPSCs: Implications for Neural Tissue Differentiation and Brain Organoid Research
Source: Cells. 2024 Mar 14;13(6):507. doi: 10.3390/cells13060507 (PMC10969360; doi:10.3390/cells13060507)
Supplement: Supplementary file 1 [file cells-13-00507-s001.zip › cells-2822855-supplementary.pdf]

cells

MDPI

Supplementary Material

Supplementary Table S 1. Gene list of affected genes as revealed by optical genome mapping in hiPSCs.

Copyright: © 2024 by the authors. Licensee MDPI, Basel, Switzerland. This article is an open access article distributed under the terms and conditions of the Creative Commons Attribution (CC BY) license (https://creativecommons.org/licenses/by/4.0/).

Chromosome	Symbol	Gene name	Gene Group
Chromosome 20. 20q11.21 duplication	DEFB115	Defensin beta 115	Defensins
	DEFB116	Defensin beta 116	Defensins
	DEFB118	Defensin beta 118	Defensins
	DEFB119	Defensin beta 119	Defensins
	DEFB121	Defensin beta 121	Defensins
	DEFB122	Defensin beta 122	Defensins
	DEFB123	Defensin beta 123	Defensins
	DEFB124	Defensin beta 124	Defensins
	REM1	RRAD and GEM like GTPase 1	RGK type GTPase family
	LINC00028	Long intergenic non-protein coding RNA 28	Long intergenic non-protein coding RNAs
	HM13	Histocompatibility minor 13	Peptidase family A22
	MCTS2	Malignant T Cell-Amplified Sequence 2, Pseudogene	
	HM13-AS1	HM13 antisense RNA 1	Antisense RNAs
	ID1	Inhibitor of DNA	Basic helix-loop-helix

		binding 1	proteins
	MIR3193	MicroRNA 3193	MicroRNAs
	COX4I2	Cytochrome c oxidase subunit 4I2	Mitochondrial complex IV: cytochrome c oxidase subunits
	**BCL2L1**	BCL2 like 1	Protein phosphatase 1 regulatory subunits. BCL2 family
	TPX2	TPX2 microtubule nucleation factor	
**Chromosome 6. 6p21.33 deletion**	PPP1R10	Protein phosphatase 1 regulatory subunit 10	Protein phosphatase 1 regulatory subunits
	MRPS18B	Mitochondrial ribosomal protein S18B	Small subunit mitochondrial ribosomal proteins.
	ATAT1	Alpha tubulin acetyltransferase 1	Lysine acetyltransferases
	C6orf136	Chromosome 6 open reading frame 136	
	DHX16	DEAH-box helicase 16	Spliceosomal Bact complex. Spliceosomal P complex. DEAH-box helicases. Spliceosomal C complex.
	PPP1R18	Protein phosphatase 1 regulatory subunit 18	Protein phosphatase 1 regulatory subunits
	NRM	Nurim (Nuclear Envelope Membrane Protein)	
	MDC1	Mediator of DNA damage checkpoint 1	
	TUBB	Tubulin beta class I	Tubulins
	FLOT1	Flotillin 1	Flotillins
	IER3	Immediate early	

		response 3	
	LINC00243	Long intergenic non-protein coding RNA 243	Long intergenic non-protein coding RNAs
	DDR1	Discoidin domain receptor tyrosine kinase 1	Receptor tyrosine kinases. CD molecules. MicroRNA protein coding host genes
	MIR4640	MicroRNA 4640	MicroRNAs
	GTF2H4	General transcription factor IIH subunit 4	Nucleotide excision repair. General transcription factor IIH complex subunits.
	VARS2	Valyl-tRNA synthetase 2, mitochondrial	Aminoacyl tRNA synthetases, Class I
	SFTA2	Surfactant associated 2	
	MUCL3	Mucin like 3	
	MUC21	Mucin 21, cell surface associated	Mucins
	MUC22	Mucin 22	Mucins
	HCG22	HLA complex group 22 (gene/pseudogene)	
	C6orf15	Chromosome 6 open reading frame 15	
	PSORS1C1	Psoriasis susceptibility 1 candidate 1	
	CDSN	Corneodesmosin	
	PSORS1C2	Psoriasis susceptibility 1 candidate 2	
	CCHCR1	Coiled-coil alpha-helical rod protein 1	
	TCF19	Transcription factor 19	PHD finger proteins
	POU5F1	POU class 5 homeobox 1	POU class homeoboxes and pseudogenes

	PSORS1C3	Psoriasis susceptibility 1 candidate 3	Long non-coding RNAs with non-systematic symbols
	HCG27	HLA complex group 27	Long non-coding RNAs with non-systematic symbols
	HLA-C	Major histocompatibility complex, class I, C	C1- set domain containing. Histocompatibility complex
	MICA	MHC class I polypeptide-related sequence A	C1-set domain containing
	HCP5	HLA Complex P5	Long non-coding RNAs with non-systematic symbols
	HCG26	HLA complex group 26	Long non-coding RNAs with non-systematic symbols
	MICB	MHC class I polypeptide-related sequence B	C1-set domain containing
	MCCD1	Mitochondrial coiled-coil domain 1	
	DDX39B	DExD-box helicase 39B	Transcriptions and export complex 1 subunits. Spliceosomal E complex. Small nucleolar RNA protein coding host genes. DEAD-box helicases.
	SNORD117	Small nucleolar RNA, C/D box 117	Small nucleolar RNAs, C/D box
	ATP6V1G2	ATPase H+ transporting V1 subunit G2	V-type ATPase subunits
	NFKBIL1	NFKB inhibitor like 1	
	LTA	Lymphotoxin alpha.	Tumor necrosis factor

			superfamily
	TNF	Tumor necrosis factor	Tumor necrosis factor superfamily
	LTB	Lymphotoxin beta	Tumor necrosis factor superfamily
	LST1	Leukocyte specific transcript 1	
	NCR3	Natural cytotoxicity triggering receptor 3	V-S domain containing CD molecules
	AIF1	Allograft inflammatory factor 1	EF-hand domain containing
	PRRC2A	Proline rich coiled-coil 2A	Small nucleolar RNA protein coding host genes. MicroRNA protein coding host genes
	SNORA38	Small nucleolar RNA, H/ACA box 38	Small nucleolar RNAs, H/ACA box
	BAG6	BAG cochaperone 6	BAG cochaperones. BAG6 complex.
	APOM	Apolipoprotein M	Apolipoproteins. Lipocalins
	C6orf47	Chromosome 6 open reading frame 47	
	GPANK1	G-patch domain and ankyrin repeats 1	G-patch domain containing. Ankyrin repeat domain containing
	CSNK2B	Casein Kinase 2 Beta	
	LY6G5C	Lymphocyte antigen 6 family member G5C	LY6/PLAUR domain containing
	ABHD16A	abhydrolase domain containing 16A, phospholipase	Abhydrolase containing domain. MicroRNA protein coding host genes.

	MIR4646	microRNA 4646	MicroRNAs
	LY6G6F	lymphocyte antigen 6 family member G6F	V-set domain containing. LY6/PLAUR domain containing
	LY6G6E	lymphocyte antigen 6 family member G6E (pseudogene)	LY6/PLAUR domain containing
	LY6G6C	lymphocyte antigen 6 family member G6C	LY6/PLAUR domain containing
	C6orf25	megakaryocyte and platelet inhibitory receptor G6b	
	DDAH2	dimethylarginine dimethylaminohydrolase 2	
	CLIC1	chloride intracellular channel 1	Chloride intracellular channels
	MSH5-SAPCD1	MSH5-SAPCD1 readthrough (NMD candidate)	
	VWA7	von Willebrand factor A domain containing 7	
	VARS1	Valine TRNA Ligase 1	Aminoacyl tRNA synthetases, Class I
	LSM2	LSM2 homolog, U6 small nuclear RNA and mRNA degradation associated	LSm proteins
	HSPA1L	heat shock protein family A (Hsp70) member 1 like	TIM23 complex. Heat shock 70kDa proteins.
	HSPA1A	heat shock protein family A (Hsp70) member 1A	TIM23 complex. Heat shock 70kDa proteins.
	HSPA1B	heat shock protein family A (Hsp70) member 1B	TIM23 complex. Heat shock 70kDa proteins.

	C6orf48	small nucleolar RNA host gene 32	Small nucleolar RNA non-coding host genes
	SNORD48	small nucleolar RNA, C/D box 48	Small nucleolar RNAs, C/D box
	SNORD52	small nucleolar RNA, C/D box 52	Small nucleolar RNAs, C/D box
	NEU1	neuraminidase 1	Neuraminidases
	SLC44A4	solute carrier family 44 member 4	Solute carriers
	EHMT2	euchromatic histone lysine methyltransferase 2	SET domain containing. Lysine methyltransferases. Ankyrin repeat domain containing.
	ZBTB12	zinc finger and BTB domain containing 12.	BTB domain containing. Zinc fingers C2H2-type.
	CFB	complement factor B	Sushi domain containing. Complement system activation components
	NELFE	negative elongation factor complex member E	RNA binding motif containing. Negative elongation factor complex members. MicroRNA protein coding host genes.
	MIR1236	microRNA 1236	MicroRNAs
	SKIV2L	SKI2 subunit of superkiller complex	Ski2 like RNA helicases. SKI complex subunits.
	DOM3Z	decapping exoribonuclease	
	STK19	serine/threonine kinase 19.	
	C4B_2	complement component 4B (Chido blood group), copy 2	

	CYP21A2	Cytochrome P450 family 21 subfamily A member 2	Cytochrome P450 family 21
	CYP21A1P	Cytochrome P450 family 21 subfamily A member 1, pseudogene	Cytochrome P450 family 21
	TNXB	Tenascin XB	Tenascins
**Chromosome 8: 8p23.3p22 deletion**	DEFB130	Defensin beta 130A	Defensins, beta
	ZNF705A	Zinc finger protein 705A	Zinc fingers C2H2-type
	LOC649352	eUbiquitin Carboxyl-Terminal Hydrolase 17-Like, pseudogene	
	DEFB109P1	Defensin beta 109A (pseudogene)	Defensins, beta
	FAM90A25P	Family with sequence similarity 90 member A25, pseudogene	
	FAM86B2	Family with sequence similarity 86 member B2	
	LOC729732	Uncharacterized LOC729732	
	LONRF1	LON peptidase N-terminal domain and ring finger 1	Ring finger proteins. MicroRNA protein coding host genes.
	MIR3926-1	microRNA 3926-1	MicroRNAs.
	MIR3926-2	microRNA 3926-2	MicroRNAs.
	LINC00681	Long intergenic non-protein coding RNA 681	Long intergenic non-protein coding RNAs
	KIAA1456	tRNA methyltransferase 9B (putative)	7BS DNA/RNA methyltransferases
	DLC1	DLC1 Rho GTPase activating protein	StAR related lipid transfer domain containing. Rho GTPase activating proteins.

	BC035181	uncharacterized LOC101930149	
	C8orf48	chromosome 8 open reading frame 48	
	SGCZ	sarcoglycan zeta	MicroRNA protein coding host genes
	Y_RNA	Ro60, Y RNA binding protein	
	MIR383	microRNA 383	MicroRNAs.
	TUSC3	Tumor suppressor candidate 3	Oligosaccharyltransferase complex subunits. Solute carriers.
	MSR1	Macrophage scavenger receptor 1	Scavenger receptors. Scavenger receptors cystein rich domain containing. CD molecules

Supplementary Table S 2. Potential CRISPR/Cas9 off-target sites identified by Cas-OFFinder for the designed gRNA.

Bulge Type	Target	Chromo-some	Position	Direction	Mis-matches	Bulge Size
X	crRNA: CGCCATGGCGACTGCGACCCCCGNGG<br>DNA: CGCCAaGGCGtCTGgGgCCCCCGCGG	Chr1	240093668	+	4	0
X	CrRNA: CGCCATGGCGACTGCGACCCCCGNGG<br>DNA: CGCCcgGGCcACcGCGACCCCCGCGG	Chr21	45541983	+	4	0
X	CrRNA: CGCCATGGCGACTGCGACCCCCGNGG<br>DNA: CGCCATGGCaACcGCGgCCtCCGGGG	Chr17	4812372	-	4	0
X	crRNA: CGCCATGGCGACTGCGACCCCCGNGG<br>DNA: CGtGGGTCaCAGTCaCCATGGCcAGG	Chr16	85729333	+	4	0

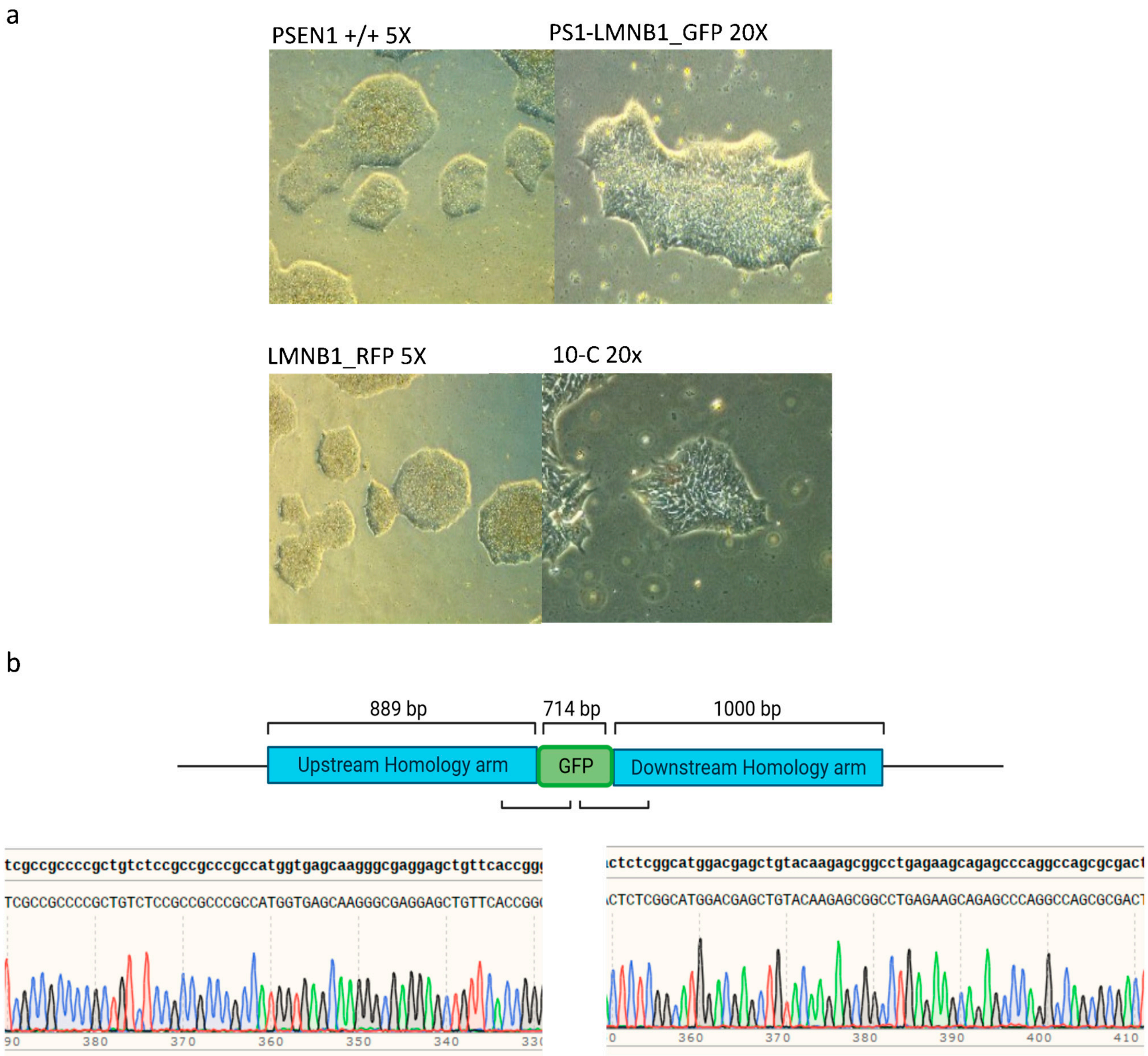

Supplementary Figure S1. Morphological and genetic assessment of CRISPR/Cas9-mediated *LMNB1* gene editing. (a) Gene-edited (PS1-LMNB1_GFP and LMNB1_RFP) and non-gene edited (10-C and PSEN1 +/+) hiPSCs colonies displayed normal morphology in culture. **(b)** Sanger sequencing data obtained after analysis of DNA of PS1-LMNB1_RFP hiPSC showing the correct knock-in of the GFP in frame with the *LMNB1* sequence.

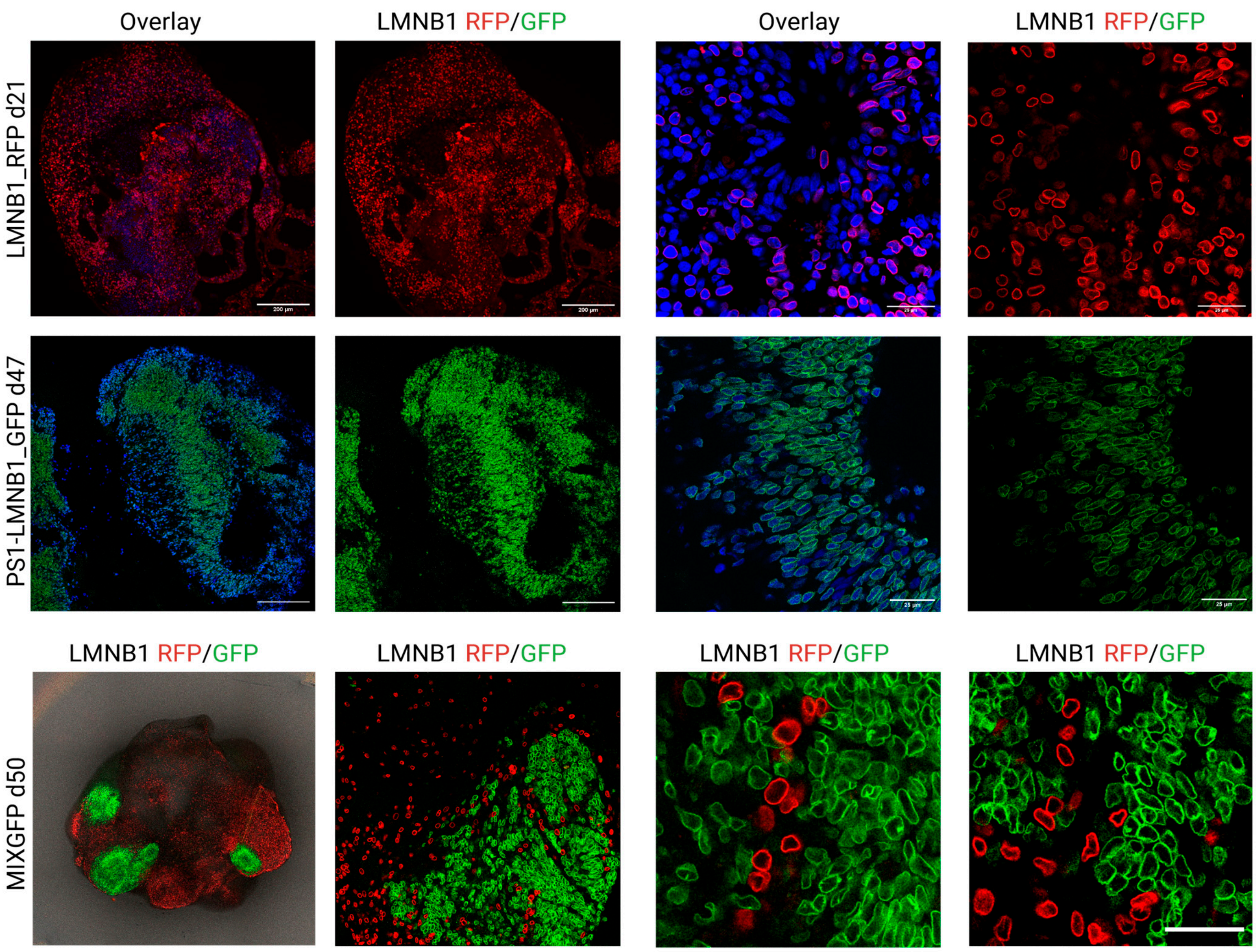

Supplementary Figure S2. Gene edited cerebral organoids stable LMNB1-tag expression. Exemplary images of LMNB_RFP (upper row), PS1-LMNB_GFP (middle row), and MIX RFP/GFP (bottom row) COs displaying LMNB1-fluorescent stable homogeneous expression. The overlay of the organoid (left) is displayed together with a detailed 100x magnification (right) where the perinuclear phenotype of the tagged lamin-B1 is discernible. Scale bar: 200 µm (overview), 25 µm (magnification).

Disclaimer/Publisher's Note: The statements, opinions and data contained in all publications are solely those of the individual author(s) and contributor(s) and not of MDPI and/or the editor(s). MDPI and/or the editor(s) disclaim responsibility for any injury to people or property resulting from any ideas, methods, instructions or products referred to in the content.
